# Reduced Cell–ECM Interactions in the EpiSC Colony Center Cause Heterogeneous Differentiation

**DOI:** 10.3390/cells12020326

**Published:** 2023-01-15

**Authors:** Kshitij Amar, Sanjoy Saha, Avishek Debnath, Chun Hung Weng, Arpan Roy, Kyu Young Han, Farhan Chowdhury

**Affiliations:** 1 School of Mechanical, Aerospace, and Materials Engineering, Southern Illinois University Carbondale, Carbondale, IL 62901, USA; 2 CREOL, The College of Optics and Photonics, University of Central Florida, Orlando, FL 32816, USA; 3 Biomedical Engineering Program, School of Electrical, Computer, and Biomedical Engineering, Southern Illinois University Carbondale, Carbondale, IL 62901, USA; 4 Materials Technology Center, Southern Illinois University Carbondale, Carbondale, IL 62901, USA

**Keywords:** EpiSCs, cell–ECM interactions, differentiation, heterogeneity, colony size

## Abstract

Mechanoregulation of cell–extracellular matrix (ECM) interactions are crucial for dictating pluripotent stem cell differentiation. However, not all pluripotent cells respond homogeneously which results in heterogeneous cell populations. When cells, such as mouse epiblast stem cells (EpiSCs), are cultured in clusters, the heterogeneity effect during differentiation is even more pronounced. While past studies implicated variations in signaling pathways to be the root cause of heterogeneity, the biophysical aspects of differentiation have not been thoroughly considered. Here, we demonstrate that the heterogeneity of EpiSC differentiation arises from differences in the colony size and varying degrees of interactions between cells within the colonies and the ECM. Confocal imaging demonstrates that cells in the colony periphery established good contact with the surface while the cells in the colony center were separated by an average of 1–2 µm from the surface. Traction force measurements of the cells within the EpiSC colonies show that peripheral cells generate large tractions while the colony center cells do not. A finite element modeling of EpiSC colonies shows that tractions generated by the cells at the colony periphery lift off the colony center preventing the colony center from undergoing differentiation. Together, our results demonstrate a biophysical regulation of heterogeneous EpiSC colony differentiation.

## 1. Introduction

From the beginning stages of a totipotent zygote, cells undergo differentiation that is analogous to a ball rolling down a landscape, passing through various pluripotent states such as inner cell mass and subsequent later-stage epiblasts. Adaptation of *in vitro* culture of a pre-implantation inner cell mass is referred to as the naïve pluripotent state, while the early post-implantation epiblasts represent the primed pluripotent state. Cells rolling down further on the differentiation cascade become multipotent progenitors and finally differentiate into adult body cells. Embryonic stem cells have been derived successfully from many species, including humans [1]. These conventional human embryonic stem cells (hESCs) are considered to be in the primed state that can be induced to more naïve-like hESC states [2,3,4]. Although mouse embryonic stem cells (mESCs) [5,6] were derived in 1981, the mouse epiblast stem cells (EpiSCs) [7,8] were established fairly recently in 2007. Interestingly, there are striking similarities between primed hESCs and mouse EpiSCs in terms of morphology, clonogenicity, global gene expression, and cytokine requirements [7,9,10]. Due to poor clonogenicity, both hESCs and mouse EpiSCs are passaged as colonies rather than single cells. For these reasons, the conventional hECSs and mouse EpiSCs are considered to be equivalent in terms of developmental stages. 

A simple and robust method for maintaining homogeneous EpiSC culture *in vitro* has been developed by introducing a Wnt inhibitor, IWP-2, in the culture medium [11]. However, upon induction of differentiation, pluripotent EpiSCs elicit a heterogeneous response [12], similar to hESCs [13,14,15]. Importantly, the underlying biophysical mechanism of such heterogeneous differentiation of EpiSCs remains largely unknown. Herein, we systematically investigated if mouse EpiSCs differentiate, similar to mouse ESCs [16], when seeded on fibronectin-coated surfaces. In addition, we determined the biophysical mechanism of heterogeneous EpiSC differentiation outcome. EpiSC colonies on fibronectin-coated surfaces applied forces on the periphery which lift off the inner colony cells, away from the fibronectin-coated surface, thus preventing differentiation in the colony center. As a result, the colony edges differentiate, but not the colony center. The larger the physical size of the colony, the more heterogeneous the cell populations become. Our systematic approach reveals an important biophysical mechanism of heterogeneous EpiSC colony differentiation on fibronectin-coated surfaces that otherwise would remain unknown.

## 2. Materials and Methods

### 2.1. EpiSC Culture and Treatments

EpiSCs, isolated from post-implantation embryos, were routinely cultured on mitotically inactivated feeders as described elsewhere [11] with Dulbecco’s Modified Eagle Medium/Nutrient Mixture F-12 basal medium (Thermo Fisher Scientific, Waltham, MA, USA; cat. # 10565018) supplemented with 15% KNOCKOUT Serum Replacement (Thermo Fisher Scientific, cat. # 10828028), 1% Penicillin Streptomycin (Thermo Fisher Scientific, #15140122), 1% MEM non-essential amino acid (Thermo Fisher Scientific, cat. #11140050), 0.1 mM 2-Mercaptoethanol (Sigma-Aldrich, St. Louis, MO, USA; cat. #M3148), 20 ng/mL Activin A (Peprotech, cat. #120-14E) and 12 ng/mL bFGF (Peprotech, Cranbury, NJ, USA; cat. #AF-100-18B), and 2 µM IWP-2 (Tocris Bioscience, Minneapolis, MN, USA; cat # 3533/10). For passaging, colonies were mildly dissociated in small clumps every other day with CTKCa dissociation buffer. The CTKCa dissociation buffer was made with Trypsin, Collagenase IV, KNOCKOUT Serum Replacement, and CaCl_2_ in PBS.

### 2.2. EpiSC Differentiation on Fibronectin

For differentiation experiments, EpiSC clusters were cultured on 1 μg/mL fibronectin-coated tissue culture dishes (Eppendorf North America, Enfield, CT, USA). The dishes were incubated with fibronectin for 1 h at 37 °C. For traction experiments, EpiSC clusters were seeded on fibronectin-coated (5 μg/mL) polyacrylamide (PAA; a mixture of acrylamide and bis-acrylamide; Bio-Rad, Hercules, CA, USA) substrates and cultured for 24 h before traction measurements.

### 2.3. Measuring Cellular Tractions on PAA Substrates

Cell traction measurements have been described elsewhere [17]. In short, images of PAA substrates, embedded with yellow-green fluorescent microbeads (0.2 μm), were captured before and after the trypsinization of cells. The displacement field was calculated based on the relative movement of beads with or without cells attached to the substrate. A Fourier Transform Traction Cytometry (FTTC)-based calculation revealed a traction map from the displacement field using the Boussinesq solution. In-house custom MATLAB codes were used for the traction force microscopy.

### 2.4. Widefield Epifluorescence Microscopy and Confocal Imaging

Phase images of differentiated and semi-differentiated colonies were obtained using an EVOS microscope (Thermo Fisher Scientific) equipped with a 10× objective. The total area and pluripotent area were calculated using ImageJ software. Fluorescent images were captured with an inverted widefield epifluorescence microscope (Leica DMi8) equipped with 20×, 40×, and 63× objectives, an Orca Flash 4.0 V2+ sCMOS camera, and a motorized stage for x-y-z position control. The microscope is also equipped with an air stream incubator for temperature control.

A multiline scanning confocal microscope [18], equipped with an Andor EMCCD camera and an NA1.4/100× Olympus objective, was used to calculate the height distribution of the periphery vs. the center of the EpiSC colonies. To fluorescently visualize the cells, EpiSCs were fixed with 4% PFA and stained with CellMask Green plasma membrane dye (Thermo Fisher Scientific).

### 2.5. Quantifying Peripheral Differentiation within the Colony

To quantify the peripheral differentiation and pluripotent center regions of the colonies, the entire colony was fitted with a circle of radius R while the peripheral differentiation regions were measured by mean radial vectors, represented by ρ. The extent of differentiation for each colony was calculated by a differentiation penetration ratio: $\frac{\rho }{R}$. For fully differentiated colonies, no pluripotent core was observed; hence, the differentiation penetration ratio was found to be 1.0. In other words, when the ratio $\frac{\rho }{R}$ was smaller than 1.0, there was a heterogeneous population present within the colony.

### 2.6. Finite Element Analysis of EpiSC Colonies

ANSYS 2019 R1 Academic solver and ANSYS 2022 R1 Student postprocessing tools were used for the analysis. EpiSC colony geometries were created based on phase-contrast colony images. For both the 1.67 kPa and the 16.7 kPa condition colony models, the total number of identified nodes was 2865, with a total number of solid elements set to 1292 after meshing. The order and mesh type were program-controlled to minimize errors. An error check was performed with “aggressive mechanical shape checking setting” and no such errors were identified by the software. The mesh size was selected within the 10–100 µm range. 

The following material properties were assigned to the finite element EpiSC colony models. An isotropic elasticity of 1 kPa and 5 kPa was assigned to the colony model for the 1.67 kPa and the 16.7 kPa PAA substrate conditions, respectively. The Poisson’s ratio was assigned to 0.49. The colonies were fixed at the bottom around the periphery, excluding the center of the colony. The forces were assigned around the edges with different magnitudes as quantified from the r.m.s. tractions. Forces were applied in “in-plane” conditions. The net force, calculated from traction experiments, was 0.32 ± 0.14 µN and 1.7 ± 0.71 µN for the 1.67 kPa and the 16.7 kPa PAA substrates, respectively.

### 2.7. Statistical Analysis

The Student’s *t*-test was carried out for statistical significance testing.

## 3. Results

### 3.1. The EpiSC Colony Size Dictates Differentiation Heterogeneity

**Figure 1:**
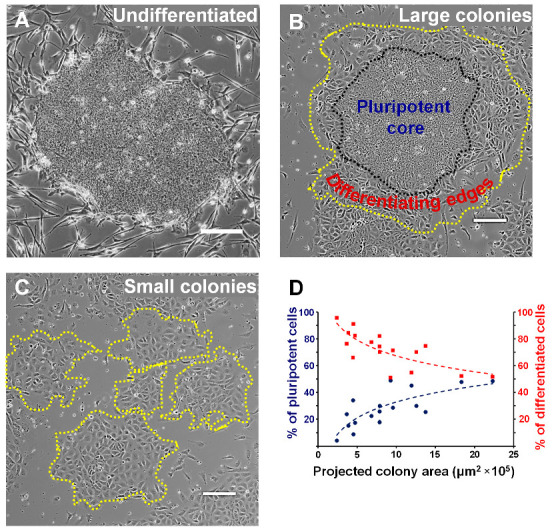
EpiSC differentiation heterogeneity is caused by the physical size of the colony. (**A**) A phase image of an EpiSC colony displays a pluripotent morphology on mitotically inactivated feeders using standard culture protocol. (**B**,**C**) Phase images of EpiSCs on fibronectin-coated surfaces reveal significant heterogeneity in EpiSC differentiation as a function of colony size. When the colony size was small, differentiation was homogeneous. As the colony size increased, pluripotency was conserved in the center while differentiation was observed only on the colony periphery. (**D**) The percentage of pluripotent and differentiated cells within the individual colonies is represented as a function of the total colony area. *n* = 17 colonies from three independent experiments. Scale bar, 200 μm.

The cloning efficiency and survival of EpiSCs after single-cell dissociation are very poor. As a result, the EpiSCs were cultured in colonies, and in every passage, the colonies were mechanically broken into small clusters. In addition, for unknown reasons, mitotically inactivated feeder coculture is reported to be indispensable for EpiSC culture success to maintain pluripotency. Following these culture conditions, the fragmented EpiSC clusters appear undifferentiated with well-defined boundaries on mitotically inactivated feeders as displayed in Figure 1A.

During differentiation, mitotically inactivated feeders were removed from the culture and the colonies were plated on surfaces coated with desired peptides, proteins, and extracellular matrix (Figure S1). The cell adhesion, spreading, and overall morphology of EpiSCs on various functionalized surfaces were observed to be significantly different. EpiSC colonies show a round spherical morphology when plated on collagen-mimicking peptide GFPGER while EpiSCs on poly-l-lysine-coated surfaces showed a highly differentiated morphology (Figure S1). The observed cell death was significant on both the GFPGER and the poly-l-lysine-coated surfaces. Interestingly, when EpiSCs were plated on E-cadherin-coated surfaces, cells appeared as single cells similar to single ESCs plated on E-cadherin-coated surfaces and may possibly lead to self-renewal as single cells [19]. On the other hand, EpiSC colonies when seeded on fibronectin-coated surfaces show a differentiated morphology with little to no cell death. In addition, fibronectin via α_5_β_1_ and α_V_β_3_ integrin signaling promotes strong adhesion and is known to induce differentiation in mouse pluripotent stem cells [16]. Therefore, we focused on studying mechanically fragmented EpiSC clusters on fibronectin-coated surfaces. To understand the differentiation effect of fibronectin alone, we used the same recipe of EpiSC culture medium that promotes self-renewal throughout all the experiments with no changes to soluble factors. Depending on the physical size of the clusters, the EpiSC colony differentiation response varied largely. For large colonies, cells on the periphery appeared to be differentiating and cells in the colony center exhibited a pluripotent morphology (Figure 1B). To clarify further, we created a Voronoi diagram to visualize the morphological difference of cell/nuclear size between the colony center and the periphery (Figure S2). Both the cell size and the nuclear size in the colony periphery appear to be larger than the colony center. Furthermore, we also labeled cytoskeletal F-actin with rhodamine-phalloidin where cells in the colony center show very little F-actin localization with no deeper F-actin cytoskeletal network (Figure S3); in contrast, the cells on the colony periphery display an extensive F-actin cytoskeletal network (Figure S3). Increased cell spreading and extensive F-actin cytoskeletal network in cells on the colony periphery indicate initiation of differentiation. As the colonies grew, the differentiation was primarily localized only to the outer periphery of the colonies while the pluripotent core remained conserved in the center. 

Interestingly, for a smaller colony size (<5 × 10^5^ μm^2^), the pluripotent core disappeared, and the differentiation efficiency increased significantly (Figure 1C,D). In other words, a smaller colony size (<5 × 10^5^ μm^2^) reduces cellular heterogeneity during differentiation. Figure 1D shows the percentage of colony cells with a pluripotent or differentiated morphology as a function of the projected colony area. When the colony size was small (~up to 5 × 10^5^ μm^2^), more than ~90% of the cells within the colony appeared differentiated (red line) while only ~10% of the cells within the colony appeared pluripotent (blue line) (Figure 1D). When the colony size progressively increased, the percentage of differentiated cells decreased with a concomitant increase in the percentage of pluripotent cells.

### 3.2. A Colony Size Threshold Exists over which Cell Differentiation Becomes Heterogeneous

**Figure 2:**
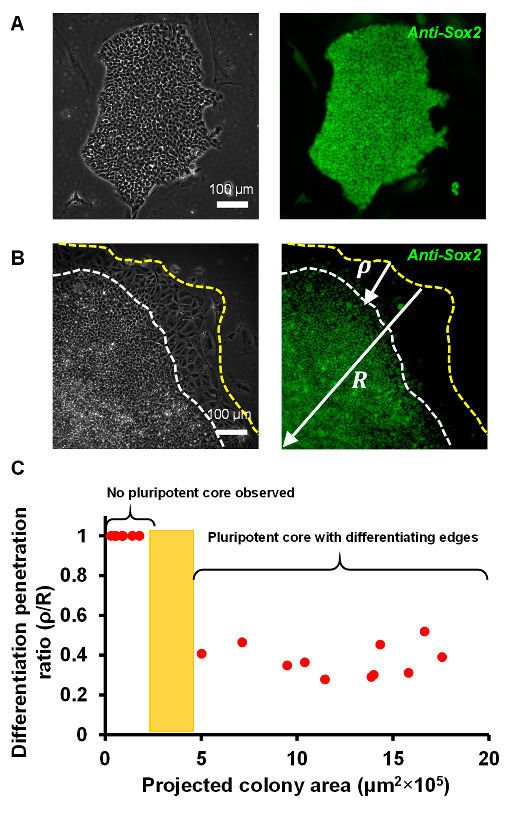
Pluripotency marker Sox2 is differentially expressed in differentiating EpiSC colonies. (**A**) Immunofluorescent labeling of pluripotency marker Sox2 in EpiSCs cultured on mitotically inactivated feeders showing no differentiating periphery. (**B**) A phase image (left) and the corresponding fluorescent image (right) show Sox2 expression in a differentiating colony. Sox2 was primarily expressed in the colony center. The outer peripheral band of the EpiSC colonies was found to be Sox2 negative. To model the differentiation effect, an average radial distance of differentiation, ρ, was defined. The radius of the colonies, R, was approximated by fitting a circle of known radius. (**C**) The scatter plot shows the ratio $\frac{\rho }{R}$ as a function of the total area of the colonies. A $\frac{\rho }{R}$ value of smaller than 1.0 indicates differentiation heterogeneity, which was found to be pronounced when the projected colony area was > 5 × 10^5^ μm^2^.

In addition to the morphological investigations, to explore the status of pluripotent markers, we carried out an immunocytochemistry assay for Sox2 expression of individual cells within the colonies. When EpiSCs were cultured on mitotically inactivated feeders, Sox2 was present uniformly throughout the colony, and there were no visible signs of differentiating edges (Figure 2A). 

In contrast, when colonies were plated on fibronectin-coated surfaces, it was evident that Sox2 was not expressed uniformly throughout the colonies. Instead, it was localized in the center of the colonies but not at the periphery of the colonies (Figure 2B). Therefore, the differentiation was primarily restricted to the periphery since the pluripotent marker Sox2 was absent, which is consistent with the morphological characterization in Figure 1. To model the extent of differentiation within the colonies as a function of the relative size of the colonies, we define a parameter, ρ, indicating the average radial distance of the differentiation band at the periphery of the colonies (Figure 2B; right). The radius, R, of the colonies was approximated by fitting a circle to the entire colony size. We define the ratio $\frac{\rho }{R}$ as the differentiation penetration ratio of the colonies and plotted the $\frac{\rho }{R}$ for all the colonies imaged as a function of the colony area as shown in Figure 2C. With larger colony size (>5 × 10^5^ μm^2^), the differentiation penetration ratio, $\frac{\rho }{R}$, remained similar around 0.3–0.5, suggesting a poor differentiation outcome. In other words, differentiation heterogeneity could be observed for colony sizes over ~5 × 10^5^ μm^2^. In contrast, when the colony size was small (<2 × 10^5^ μm^2^), the differentiation penetration ratio, $\frac{\rho }{R\;}$, was found to be 1.0, indicating a 100% differentiation within the colony. We did not observe any colony sizes between 2 × 10^5^ and 5 × 10^5^ μm^2^ in these experiments, and therefore, in the transition zone (Figure 2C, yellow bar) the differentiation penetration ratio (i.e., presence of heterogeneity) could not be evaluated. Further investigation is needed to elucidate the heterogeneity pattern within the transition zone; nevertheless, a clear threshold of colony size (>5 × 10^5^ μm^2^) could be observed, over which, the cell differentiation becomes heterogeneous.

### 3.3. Reduced Cell–ECM Contact in the Colony Center Prevents Differentiation

**Figure 3:**
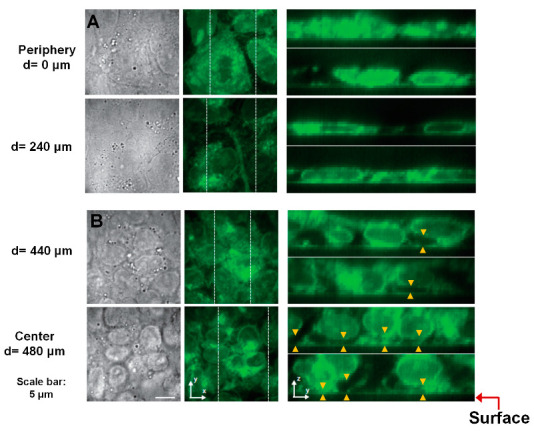
3D confocal microscopy images of EpiSC colonies reveal a significant gap between cells and the fibronectin-coated surface in the colony center. Brightfield (left) and fluorescence images of EpiSC colonies in the x–y (middle) and y–z planes (right) at different locations are shown. The y–z sections were taken from the 3D image stacks indicated by white dashed lines in the x–y sections. Cells were stained with CellMask green plasma membrane dye. 3D image stacks of fluorescence images were obtained by multiline scanning confocal microscope. The brightfield images were obtained 2 μm above the surface. A red arrow indicates the surface. Cells on the periphery establish good contact with fibronectin that promotes differentiation (**A**). In contrast, cells in the colony center show significant gaps between the cells and the surface, thereby decreasing cell–ECM interactions ((**B**); arrowheads). Cells in the colony center have been lifted away from the surface by 1–2 μm, on average, and in some places by >3 μm. Scale bar, 5 μm.

Since cells in the periphery were differentiating but not cells in the colony center, we postulated that there might be a varying degree of cell–ECM contact within the colonies which gives rise to the differentiation heterogeneity. To determine the extent of cell–ECM contacts within the colonies, we imaged the colonies in 3D using confocal microscopy at varying distances from the periphery to the center of the colonies. As before, EpiSC colonies on fibronectin-coated surfaces were fixed and stained with CellMask green plasma membrane dye. Figure 3 shows the brightfield and corresponding fluorescent cell boundary images at different distances from the colony periphery. In Figure 3A, the first row displays brightfield images (left panel) of well spread out cells within the colony at the periphery (d = 0 μm) with corresponding fluorescent images in the x–y plane (middle panel). The y–z plane images are shown in the right panel for the two white dotted lines in the x–y plane. From these images, it is evident that strong cell–ECM contact exists at the periphery. At a distance of 240 μm from the periphery (d = 240 μm), similar good contact between the cell and ECM was observed. However, close to the colony center, at a distance of 440 μm from the periphery (d = 440 μm), cells were not well spread out, appeared more rounded, and showed gaps between cells and the surface (Figure 3B; arrowheads). At the colony center (d = 480 μm), the gaps between the cells and surface are even more pronounced (Figure 3B, bottom row; arrowheads), thus creating reduced cell–ECM contacts and preventing fibronectin on the surface to induce cell differentiation. Furthermore, to validate a difference in cell–ECM contacts between the periphery and the colony center, we fixed and stained EpiSC colonies with a paxillin antibody. Figure S4 shows positive focal adhesion contact in the colony periphery but not in the colony center.

### 3.4. Traction Stresses at the Colony Periphery Lead to Heterogeneous Differentiation

**Figure 4:**
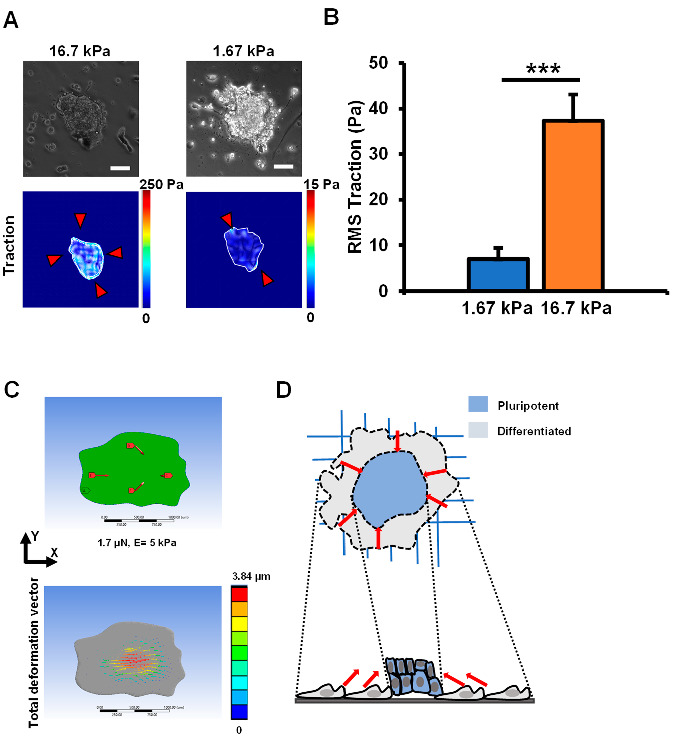
Elevated traction stresses at the EpiSC colony periphery lift the colony center from the surface. (**A**) Representative images of EpiSC colonies cultured on 1.67 kPa and 16.7 kPa PAA substrates and their corresponding traction maps are shown here. Scale bar, 50 μm. (**B**) Data summary of EpiSC colony r.m.s. tractions on 1.67 kPa and 16.7 kPa substrates is presented here. Data represent mean ± s.e.m. from three independent experiments. *n* = 16 for 1.67 kPa and *n* = 10 for 16.7 kPa PAA substrates. Student’s two-tailed t-test was used to conduct statistical analysis for the two groups (***, *p* = 0.00037). (**C**) ANSYS model was created to represent a pseudo-colony morphology. F = σ_r.m.s._ * Area was used to calculate force from the traction maps. F = 1.7 µN for the 16.7 kPa PAA gel condition was applied to the model in an in-plane configuration at ~200 µm away from the periphery. (**D**) A proposed model of EpiSC colony differentiation shows that the colony center is elevated due to the force applied on the periphery, which leads to the maintenance of self-renewal of cells in the colony center.

Since a differential cell–ECM interaction was observed for the colony periphery and the center, we next investigated the tractions generated within the colonies. Based on reduced cell–ECM contact observation, we speculated that traction stresses would also vary between the colony periphery and the center. Traction stresses are an important parameter to determine stress-mediated cell differentiation of pluripotent cells [20]. The overall role of forces/stresses and force-mediated signaling in regulating the fate decisions of stem cells has been reviewed elsewhere in detail [21]. As predicted, the traction magnitude at the basal colony surface was found to be high at the periphery and low at the colony center (Figure 4A), which is consistent with the 3D confocal imaging data in Figure 3 and Figure S4. EpiSCs plated on 16.7 kPa substrate exhibited a root mean square (r.m.s.) traction of ~40 Pa (Figure 4B). To reduce the traction in the cell periphery, we used the Y27632 compound, an inhibitor of Rho-kinase (ROCK). With Y27632 compound treatment, the r.m.s. traction of the EpiSC colonies was reduced to ~17 Pa (Figure S5). In addition, previous studies have shown that Arp2/3 was present on the leading edge which influences leading-edge protrusion and motility [22]. Therefore, we focused on inhibiting the Arp2/3 complex that may be responsible for the nucleation of the branched actin cone dynamics at the colony periphery. We tested with two small molecule inhibitors of the Arp2/3 complex, namely, CK666 and CK869 [23]. When we treated EpiSC colonies with CK666, the r.m.s. traction magnitude at the basal surface was found to be ~20 Pa, similar to the treatment with the Y27632 compound (Figure S5). With CK869 treatment, the reduction of traction was more pronounced (~10 Pa) but not statistically different from the Y27632 and CK666 treatment groups (Figure S5). Next, we plated EpiSCs on 10-fold softer substrates (1.67 kPa), and the r.m.s. traction was found to be significantly lower at ~10 Pa. Unlike the colonies on the 16.7 kPa substrates which generated uniform traction around the colony periphery, the colonies on the 1.67 kPa substrates generated occasional peak tractions of low magnitude around the colony periphery. Interestingly, the shape of the colonies was very similar, thus suggesting that EpiSC colonies were able to sense substrate rigidity and accordingly modulate their basal tractions. In comparison, pluripotent mouse embryonic stem cell colonies also exhibited r.m.s. traction between 40 and 50 Pa depending on the substrate stiffness [20]. However, mouse embryonic stem cell colonies did not exhibit differential tractions between the colony periphery and the center. Therefore, the elevated tractions in the colony periphery in combination with reduced traction in the colony center is a unique feature of EpiSC colonies.

### 3.5. A Putative Model Explaining Cell Heterogeneity during EpiSC Colony Differentiation

To simulate whether the forces at the colony periphery are causing the EpiSC center to lift off, we used a finite element analysis (FEA) model to investigate the deformation in the z-direction throughout the colony. We developed an EpiSC-specific FEA model with an arbitrarily assigned elasticity of 5 kPa. As cells were plated on 16.7 kPa substrate, it is assumed that cells within the EpiSC colonies will tune their intrinsic stiffness to match that of their underlying substrate. However, cell stiffness matching with the substrate stiffness begins to plateau at around 20 kPa with a measured cell stiffness of ~5 kPa [24]. Therefore, the assigned elasticity of 5 kPa to EpiSCs on a 16.7 kPa substrate is a reasonable estimation. The modeled colony images were acquired from phase-contrast microscopy images. For assigning the force boundary conditions, inward forces of 1.7 μN were applied on the periphery of the colony as indicated in Figure 4C. Furthermore, the colony was fixed at the bottom, around the periphery, excluding the colony center. We justify this boundary condition based on the experimental traction force measurements (Figure 4), confocal imaging (Figure 3), and focal adhesions as visualized by paxillin staining (Figure S4). After running the FEA simulation, a heatmap of the total deformation vector throughout the colony is presented in Figure 4C. The peak deformation in the colony center was found to be ~3.8 μm in the colony center (Figure 4C). As expected, the z-deformation gradually diminished towards the periphery. Next, we analyzed the peak z-deformation in the colony center for an EpiSC colony on a 1.67 kPa substrate. The assigned elasticity was 1 kPa, and forces applied on the colony periphery were set to 0.32 μN. As before, we followed the substrate stiffness matching trend to assign an elasticity of 1 kPa for EpiSC colonies on a 1.67 kPa substrate [24]. In addition, the applied forces were based on traction force measurements on 1.67 kPa substrates. The total deformation vector shows distortion in the colony center with a peak deformation of ~3.6 μm, similar to EpiSC colonies on 16.7 kPa substrates (Figure S6). Regardless of the substrate stiffness and force boundary conditions, it is clear that the center of the EpiSC colony will be elevated, which is consistent with the confocal microscopy data as shown in Figure 3. Based on these findings, we propose a putative model for EpiSC colony differentiation as shown in Figure 4D. Cells in the EpiSC colony periphery establish strong contact with the ECM and generate inward traction along with the initiation of the differentiation process. The generated inward traction at the colony periphery elevates the colony center. As a result, the colony center maintains pluripotency, thus resulting in heterogeneous differentiation within the same EpiSC colony.

## 4. Discussion

In this study, we investigated how EpiSCs as a colony respond to mechanical forces when allowed to generate endogenous forces during differentiation. We found that only the cells on the colony periphery establish good contact with the fibronectin-coated surface and undergo differentiation but not the cells in the colony center. Our study showed that large colonies generate traction forces that elevate the colony center and support the maintenance of self-renewal in the colony center. It is to be noted that fibronectin alone was sufficient to initiate differentiation, and no differentiation induction medium was used during this study. When cells establish good contact with fibronectin-coated surfaces and are allowed to generate mechanical forces, they undergo cell differentiation. The underlying mechanism is further discussed elsewhere [25]. Past studies have demonstrated that mouse mESCs differentiate in the presence of fibronectin or synthetic Arg-Gly-Asp (RGD) peptides [16,26]. In contrast, when mechanical forces were dominated by cell–cell adhesion (e.g., via E-cadherins), as opposed to cell–ECM (e.g., fibronectin) interactions, the cell fate was directed towards self-renewal [19,27], and any interference of E-cadherin would cause morphological changes and differentiation [28,29]. EpiSCs on the colony center may be interacting with neighboring cells via E-cadherins, which translates into self-renewal, while EpiSCs on the colony periphery are favoring differentiation via cell–ECM (fibronectin) interactions. Although it is not the focus of this paper, our preliminary studies regarding EpiSC adhesion on E-cadherin indicate the possibility of pluripotency maintenance as single cells rather than colonies, which will be investigated in the future. 

It is not quite clear if the dominance of force signaling in cell–cell vs. cell–ECM pathways exists in vivo during mammalian embryogenesis; however, the current evidence seems to support this notion. From totipotent zygote to downstream pluripotent stages, the role of mechanical force via E-cadherins can be critical since E-cadherin-null mouse embryos only form loose cell aggregates, thus failing to develop into animals [30]. In the later stages of the blastocyst development, when fibronectin is first expressed in the embryo, there may be a switch in mechanical force transduction pathways which leads to force transduction dominance via cell–ECM instead of cell–cell interactions. Whether it is cell–cell (via E-cadherins) or cell–ECM (via integrin–fibronectin) interactions, the actomyosin forces can be presumed to play very important roles in embryogenesis. In our study, we may be observing similar events in the EpiSC center vs. the periphery.

Fibronectin has an opposite role in the fate decisions of mouse vs. human pluripotent stem cells. Although mouse EpiSCs share similarities with conventional hESCs in many ways, the activation of mechanotransduction pathways via fibronectin for cell fate decisions of mouse vs. human pluripotent stem cells is very different [31]. Fibronectin and its binding partners such as α_5_β_1_ integrins are essential in mouse embryonic development and serve to support mesenchymal cell migration during embryogenesis [32,33]. Embryos that are null for fibronectin or α_5_β_1_ integrins show distinct defects in mesodermal development [34,35]. In addition, fibronectin or fibronectin-mimicking RGD tripeptides-based mechanotransduction studies showed initiation of cell differentiation of mouse ESCs [21]. Furthermore, fibronectin has been shown to activate the ERK/ MAPK pathway to induce differentiation in mouse ESCs [16]. In contrast, fibronectin has been shown to support the long-term self-renewal of hESCs [36]. In addition, a gelatinous protein mixture (commonly known as Matrigel) containing a trace amount of fibronectin has been widely used for the maintenance of hESC self-renewal. Therefore, there is an opposing biological role of fibronectin in mouse pluripotent cells vs. conventional hESCs. In this study, we show that mouse EpiSCs, similar to mESCs, differentiate on fibronectin-coated surfaces. However, EpiSC differentiation, particularly for large colonies, was restricted to the peripheral cells only. The generated tractions at the periphery elevate the colony center preventing cell differentiation. Therefore, cell heterogeneity can be observed in EpiSC colonies during differentiation.

The regulation of endogenous forces in hESCs after cell dissociation is very critical. The cortical tension generated by actomyosin contractility in hESCs induces apoptosis, but the inhibition of Rho-associated protein kinase (ROCK) relieves this reliance [37]. We wondered if the ROCK inhibitor can downregulate traction in the colony periphery. With the ROCK inhibition, EpiSCs minimized traction generation in the periphery and, subsequently, the entire colony made contact although the r.m.s. tractions were 2.5 times lower than the control group. Similarly, when cells were treated with Arp2/3 complex inhibitors, namely, CK666 and CK869 [23,38], we successfully reduced the generation of the peripheral tractions. However, the overall r.m.s. tractions were downregulated, similar to the ROCK inhibitor. Mechanical forces, whether endogenously generated or externally applied via integrin pathways, are known to play an important role. If EpiSCs were allowed to develop uniform tractions throughout the colony, it may result in cell differentiation homogeneity. However, with downregulated r.m.s. tractions, the overall cell differentiation outcome is currently not known and is beyond the scope of this work. In the future, various concentrations of ROCK and Arp2/3 complex inhibitors will be tested, and the differentiation outcome will be evaluated. In addition to fibronectin, the influence of laminin or type-IV collagen may be investigated to determine EpiSC colony response and subsequent cell lineage commitments.

## 5. Conclusions

Taken together, we show that EpiSCs differentiate on fibronectin-coated surfaces, similar to mouse ESCs, without any addition of differentiation-inducing soluble factors. Importantly, we identified the biophysical mechanism of cellular heterogeneity during EpiSC colony differentiation―the physical size of the colony is what dictates differential interactions with the fibronectin-coated surface and triggers varying differentiation responses. With large EpiSC colonies, the colony periphery establishes good contact with the fibronectin-coated surface and, thus, generates inward traction force around the periphery. Tractions generated in the colony periphery lift off the colony center. As a result, only the colony periphery undergoes differentiation but not the colony center. In contrast, for small colonies, the differentiation efficiency was found to be very high with little to no observed differentiation heterogeneity.

## Data Availability

The data are available from the corresponding author upon request.

## Supplementary Materials

The following supporting information can be downloaded at: https://www.mdpi.com/article/10.3390/cells12020326/s1. Figure S1: Morphological differences of EpiSCs cultured on GFPGER (collagen mimetic peptide), E-cadherin, fibronectin, and poly-L-Lysine. Figure S2: A qualitative analysis of cell size in the colony periphery vs. colony center using the Voronoi tessellation method. Figure S3: F-actin organization in the colony periphery vs. the colony center. Figure S4: Confocal microscopy images of EpiSC colony stained with anti-paxillin antibody shows focal adhesion formation in the colony periphery but not in the colony center. Figure S5: Various pharmacological interventions show downregulation of r.m.s. tractions in EpiSCs. Figure S6: ANSYS modeling of an EpiSC colony on 1.67 kPa substrate shows elevation of the colony center due to the force application around the colony periphery. References [20,39,40] are cited in supplementary materials.
