# Supplementary material for: Reduced Cell–ECM Interactions in the EpiSC Colony Center Cause Heterogeneous Differentiation
Source: Cells. 2023 Jan 15;12(2):326. doi: 10.3390/cells12020326 (PMC9857087; doi:10.3390/cells12020326)
Supplement: Supplementary file 1 [file cells-12-00326-s001.zip › cells-2142419-supplementary.pdf]

Supplemental Information for "Reduced Cell-ECM Interactions in the EpiSC Colony Center Cause Heterogeneous Differentiation"

Kshitij Amar [1,†], Sanjoy Saha [1], Avishek Debnath [1], Chun Hung Weng [2], Arpan Roy [1], Kyu Young Han [2] and Farhan Chowdhury [1,3,4,*]

1 School of Mechanical, Aerospace, and Materials Engineering, Southern Illinois University Carbondale, Carbondale, IL 62901, USA
2 CREOL, The College of Optics and Photonics, University of Central Florida, FL 32816, USA
3 Biomedical Engineering Program, School of Electrical, Computer, and Biomedical Engineering, Southern Illinois University Carbondale, Carbondale, IL 62901, USA
4 Materials Technology Center, Southern Illinois University Carbondale, Carbondale, IL 62901, USA
† Present Address: Department of Mechanical Science and Engineering, University of Illinois at Urbana-Champaign, Urbana, IL 61801, USA
* Correspondence: farhan.chowdhury@siu.edu

This PDF file includes additional

- Experimental Procedures [Pg. 2]
- Supplementary Figures [Pg. 4-9]
- References [Pg. 10]

Supplementary Experimental Procedures

Fabrication of polyacrylamide substrates

Thin (~70 μm thickness) polyacrylamide substrates were made as described before [1–3]. The elastic Young's modulus of the polyacrylamide substrates used here was 1.67 kPa (3% acrylamide, 0.225% bis-acrylamide)) and 16.7 kPa (10% acrylamide, 0.15% bis-acrylamide). Yellow-green fluorescent micron-sized beads (0.2 μm; Thermo Fisher Scientific cat. #F8811) were embedded on the substrates for traction measurements.

Immunocytochemistry

EpiSCs were cultured on 35 mm glass-bottom dishes (Cellvis; cat. # D35-14-0-N) coated with fibronectin. Cells were fixed using 4% paraformaldehyde (PFA) for 10 minutes at room

temperature. To block the surface, we used a blocking buffer made with PBS containing 0.5% Triton-X and 3% BSA. After blocking the surface for 1 hour at room temperature, the fixed cells were incubated with a primary Rabbit polyclonal anti-SOX2 antibody (Abcam) for 1.5 hours. After three washes with blocking buffer (5 min each time), cells were incubated with anti-rabbit goat antibodies secondary antibodies tagged with Cy3 fluorophores for 1 hour. The samples were washed three times (5 min each). Following the wash, samples were mounted with Prolong Gold Antifade reagent (Thermo Fisher Scientific). Imaging was conducted using a 20X objective on a Leica DMI8 epifluorescence microscope.

For Paxillin staining, monoclonal Anti-Paxillin primary antibody (Abcam) was used with cells fixed with 4% PFA for 10 minutes. Primary antibody treatment was conducted for 1.5 hours, followed by 2X PBS wash. Secondary antibody, conjugated with Alexa Fluor 594 (Abcam), was incubated for 1 hour. Cells were washed with 2X with PBS and then the coverslip was mounted with Diamond Anti-fade mountant (Thermo-Fisher). Images were taken on a Leica SP8 confocal with an excitation laser line of 561 nm. Images were acquired using LASX software and prepared using ImageJ.

Supplementary Figures

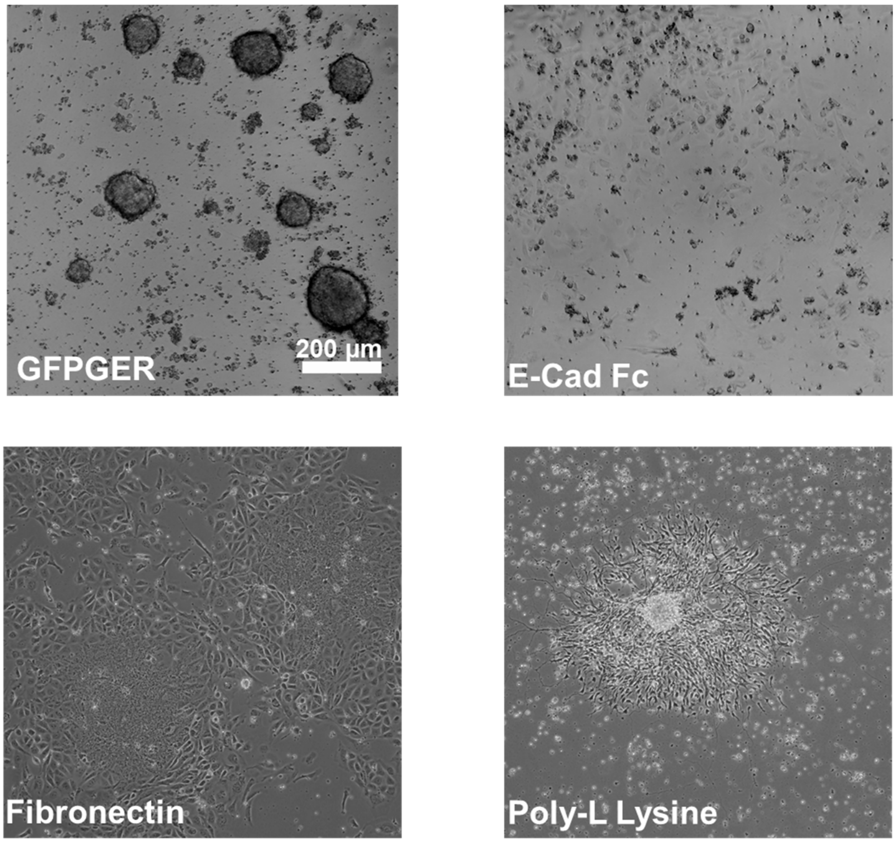

Figure S1. Morphological differences of EpiSCs cultured on GFPGER (collagen mimetic peptide), E-cadherin, fibronectin, and poly-L-Lysine. Note that cells grow well on fibronectin coated surfaces but exhibit differentiated morphology. There is a distinct morphological difference of EpiSCs when cultured on other functionalized surfaces.

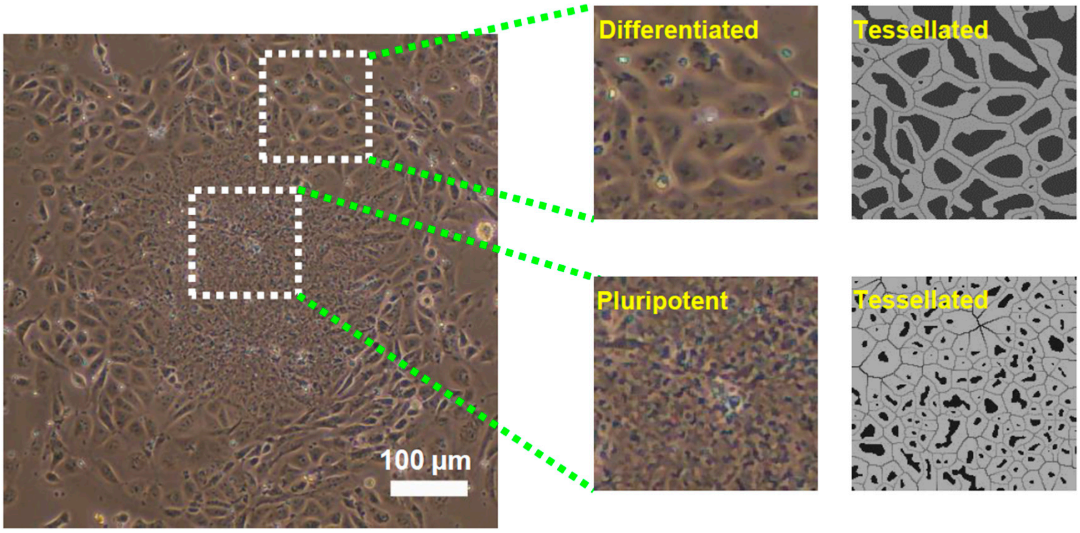

Figure S2. A qualitative analysis of cell size in the colony periphery vs. colony center using the Voronoi tessellation method. The Voronoi diagrams illustrate that both cell and the nuclear size in the colony periphery is larger than the cells in the colony center. Higher cell spreading on the periphery on the fibronectin coated surface indicates the initiation of differentiation.

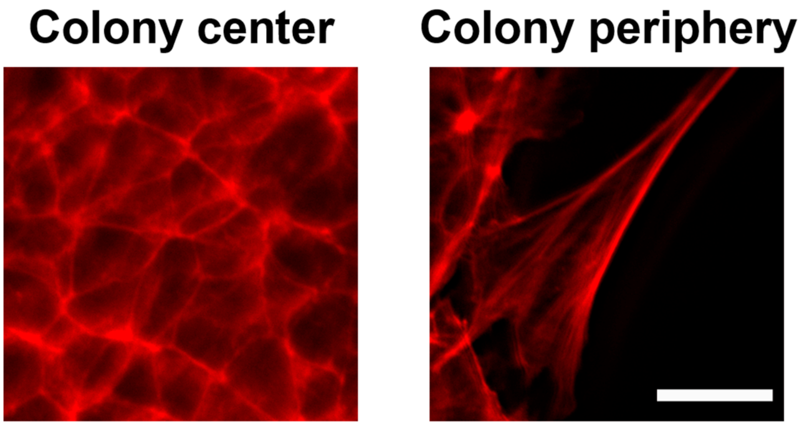

Figure S3. F-actin organization in the colony periphery vs. the colony center. Rhodamine-phalloidin based F-actin staining reveals an extensive F-actin cytoskeletal network in cells on the colony periphery (right) compared to cells in the colony center (left). Scale bar, 20 µm.

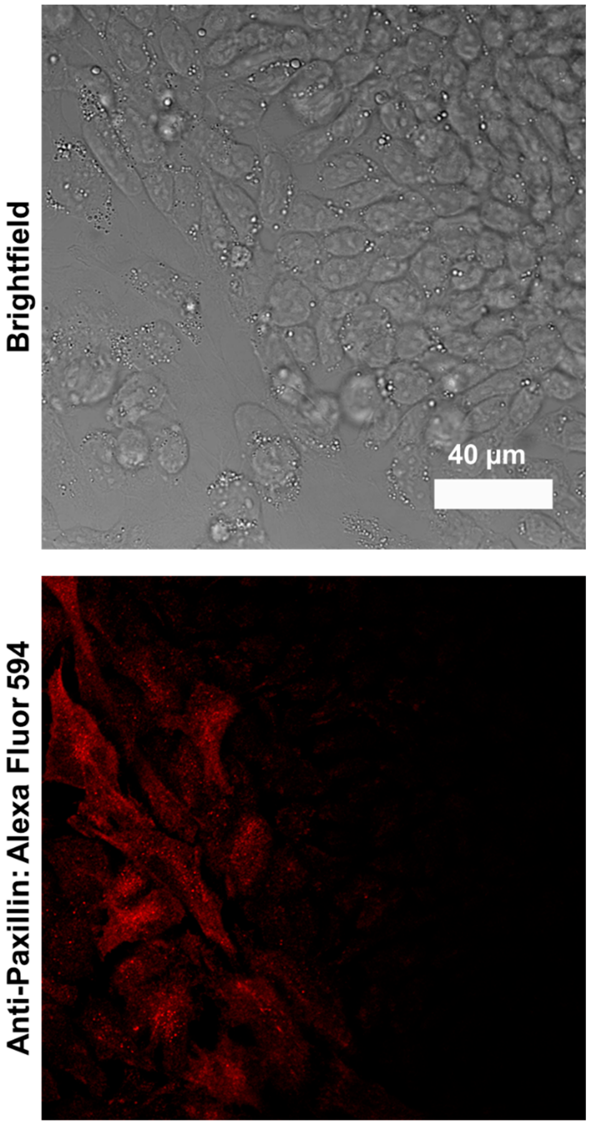

Figure S4. Confocal microscopy images of EpiSC colony stained with anti-paxillin antibody shows focal adhesion formation in the colony periphery but not in the colony center. 1:100 dilution of Anti-Paxillin primary antibody and 1:500 dilution of secondary antibody were used.

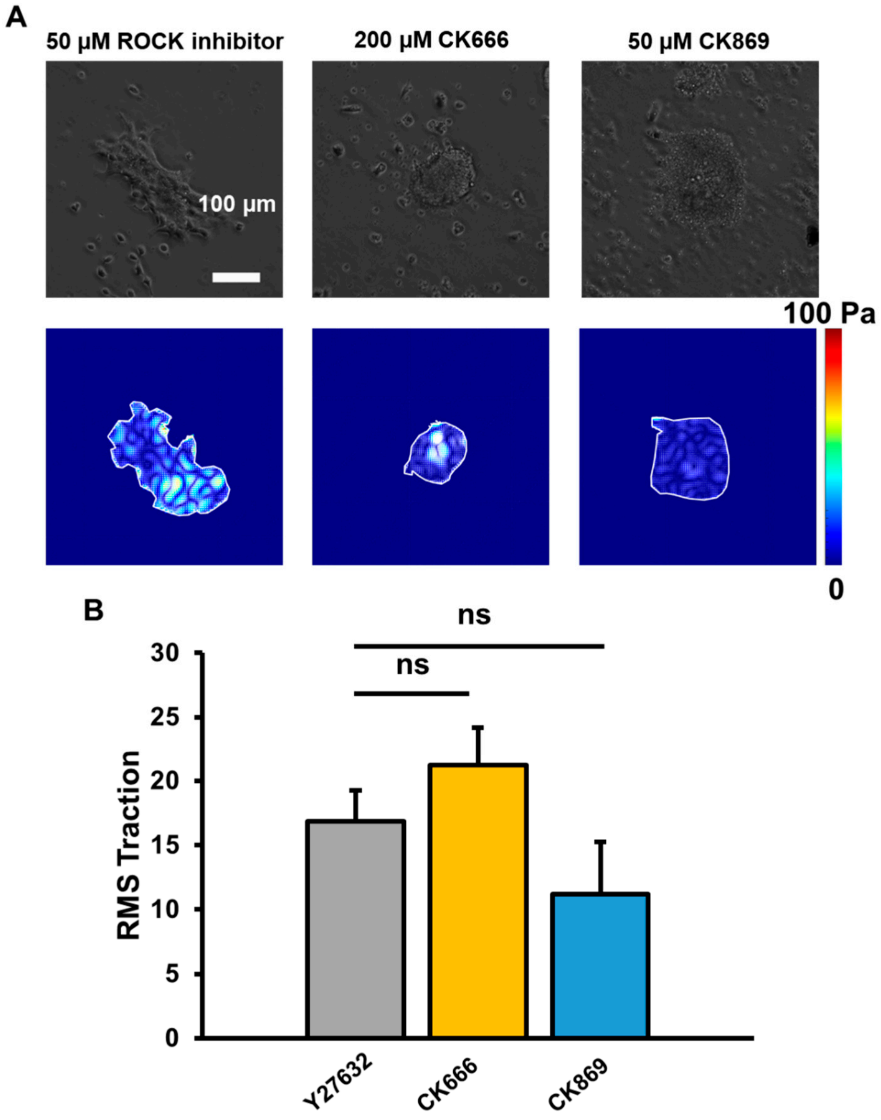

Figure S5. Various pharmacological interventions show downregulation of r.m.s. tractions in EpiSCs. A) Pluripotent EpiSCs were treated with 50 µM Y27632 ROCK inhibitor, 200 µM CK666 and 50 µM CK869 before plating cells on 16.7 kPa polyacrylamide gels. Traction measurements were made 24 hours after treatment. B) RMS traction of different drug treated cells is shown as mean ± s.e.m. n = 5, 6 and 3 cells for ROCK inhibitor, CK666, and CK869 respectively. ns = not statistically different.

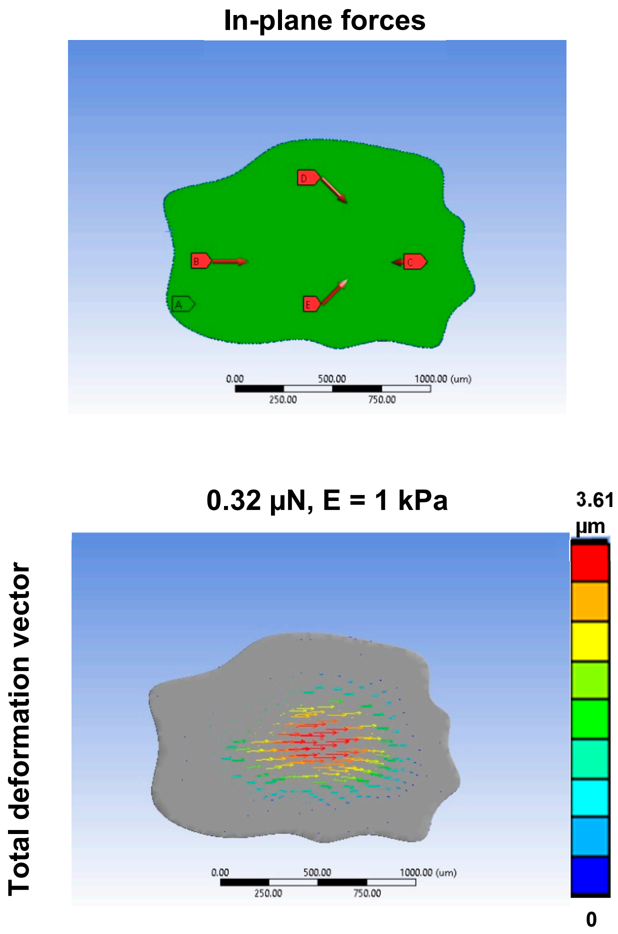

Figure S6. ANSYS modeling of an EpiSC colony on 1.67 kPa substrate shows elevation of the colony center due to the force application around the colony periphery. The forces applied are F = 0.32 µN in a 2D and 3D format. Forces were calculated using F = r.m.s. traction * Area of the colony. The young's modulus of the colony was chosen to be 1 kPa based on substrate stiffness matching model. The total deformation vector with corresponding scale are shown.

References

1. Chowdhury, F.; Li, Y.; Poh, Y.-C.; Yokohama-Tamaki, T.; Wang, N.; Tanaka, T.S. Soft Substrates Promote Homogeneous Self-Renewal of Embryonic Stem Cells via Downregulating Cell-Matrix Tractions. *PLoS ONE* **2010**, *5*, doi:10.1371/journal.pone.0015655.
2. Tse, J.R.; Engler, A.J. Preparation of Hydrogel Substrates with Tunable Mechanical Properties. *Current Protocols in Cell Biology* **2010**, *47*, doi:10.1002/0471143030.cb1016s47.
3. Engler, A.; Bacakova, L.; Newman, C.; Hategan, A.; Griffin, M.; Discher, D. Substrate Compliance versus Ligand Density in Cell on Gel Responses. *Biophysical Journal* **2004**, *86*, 617-628, doi:10.1016/s0006-3495(04)74140-5.
